# 2295. Documented SARS-CoV-2 Transmission Events Between Patients and Hospital Staff

**DOI:** 10.1093/ofid/ofad500.1917

**Published:** 2023-11-27

**Authors:** Alison Han, Heike Bailin, Jennifer Kwan, Michael Bell, Brooke K Decker, Tara Palmore

**Affiliations:** National Institutes of Health, Bethesda, Maryland; NIH/OD/ORS/OMS, Bethesda, Maryland; NIH/NIAID, Bethesda, Maryland; NIH/OD/ORS/OMS, Bethesda, Maryland; VA Pittsburgh Healthcare System, Pittsburgh, Pennsylvania; George Washington School of Medicine & Health Sciences, DC

## Abstract

**Background:**

Non-Pharmaceutical Interventions (NPIs) were critical for the protection of healthcare personnel (HCP) from SARS-CoV-2 infection throughout the pandemic. Our review of several instances of patient-to-HCP, HCP-to-patient, and HCP-to-HCP spread showed that transmission events were primarily associated with not wearing masks.

**Methods:**

Risk of transmission was assessed from tracing interviews. Possible or probable workplace-acquired COVID-19 was assessed in cases with high-risk exposures to infectious patients or staff and prolonged contact and plausible exposure timeline. HCP with dual masking between case and contact (both wearing a tight-fitting medical mask) were considered at no or low risk for exposure. Whole genome sequencing information was available for suitable samples in a subset of case studies.

**Results:**

From March 3, 2020-March 31, 2023, there were 3,513 SARS-CoV-2 infections among healthcare workers. Of the total known HCP infections, 249 cases were possibly or probably associated with the workplace. An additional 25 cases with suspected workplace transmission were found to have low-risk contacts at work, but competing high-risk exposures outside of work. Stratifying by transmission vector dynamic, 146 (58.6%) had a documented epidemiologic link to another HCP within the workplace. Five (2.0%) HCP cases had a documented epidemiologic link to a patient and 1 (0.4%) had a documented epidemiologic link to another HCP and a patient. In 2 instances HCP were epidemiologically linked to a subsequent patient infection. Whole viral genome sequencing of suitable samples refuted workplace transmission in some cases and substantiated the linkage from tracing data in others.

**Conclusion:**

After accounting for reported exposure sources, workplace transmission rates were extremely low in instances where NPIs were applied. Suspected transmission events were associated with instances where masking was relaxed between co-workers or in patient care instances where masking was not feasible by the patient.

**Disclosures:**

**Tara Palmore, MD**, Abbvie: Grant/Research Support|Gilead: Grant/Research Support|Rigel: Grant/Research Support

